# Taxonomic identification and life cycle comparison of two populations of the monostromatic green algae *Monostroma nitidum*

**DOI:** 10.1002/ece3.11424

**Published:** 2024-05-21

**Authors:** Jiawei Liao, Sipan Wang, Kun Lin, Yongjian Huang, Xinyi Chen, Rong Xin, Youyou Guo, Enyi Xie

**Affiliations:** ^1^ Fishery College Guangdong Ocean University Zhanjiang China

**Keywords:** evolution, life cycle, molecular analysis, *Monostroma nitidum*, taxonomy

## Abstract

*Monostroma nitidum*, a monostromatic green algae (MGA) with high economic value, is distributed worldwide. Life cycle often serves as a fundamental criterion for taxonomic classification. Most researchers consider the life cycle of *M. nitidum* to involve dimorphic alternation of generations, although the possibility of a monomorphic asexual life cycle remains unclear. In this study, *tufA* and 18S rDNA sequences were employed as molecular markers, complemented by morphological analysis, to classify and identify MGA in two distinct habitats: Hailing Island reefs (YJ) and Naozhou Island reefs (ZJ). The results of *tufA* and 18S rDNA sequence analysis revealed that all samples from YJ and ZJ clustered to the same branch (*M. nitidum* clade) with high bootstrap support and genetic distances of less than 0.000 and 0.005, respectively. However, morphological observations indicated significant differences in the external morphology of the YJ and ZJ samples, although both initially exhibited a filament‐blade form during early development. The life cycle of the ZJ samples exhibited typical dimorphic alternation of generations, whereas the YJ samples only produced biflagellate asexual gametes with negative phototaxis. Gametes of the YJ samples directly developed into new gametophytes without undergoing the sporophyte stage. Consequently, the YJ and ZJ samples were classified as monomorphic asexual and dimorphic sexual *M. nitidum*, respectively. These findings provide evidence supporting the monomorphic asexual life cycle of *M. nitidum* for the classification of MGA.

## Graphical abstract

This study employed molecular markers and morphological studies of two populations of *Monostroma nitidum* collected from YJ and ZJ. The results revealed no obvious differences in *tufA* and 18S rDNA sequences between the two populations, although there were extremely significant differences in morphological characteristics. *M. nitidum* collected from YJ exhibited only a monomorphic asexual life cycle, while samples collected from ZJ followed the typical dimorphic alternation of generations. The results of this study demonstrate that an asexual life cycle is insufficient as the only classification of monostromatic green algae at the genus level. *M. nitidum* has both sexual and asexual life cycles. The emergence of asexual *M. nitidum* is the result of environmental selection, they are secondary and have lost the capacity for sexual reproduction for the second time over a long evolutionary period.

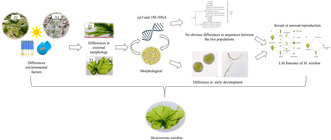

## 1 INTRODUCTION

Marine macroalgae are photoautotrophic organisms without roots, stems, leaves, or embryos. To date, more than 140 kinds of edible marine macroalgae rich in minerals, vitamins, and dietary fiber have been described (Klnc et al., 2013; Pereira, 2011), Marine macroalgae are consumed as traditional foods in Asian countries (Mouritsen et al., 2019) and used as important sources of raw materials for production of pharmaceuticals, chemicals, and animal feed (Leandro et al., 2019). In addition, marine macroalgae has continued to attract increasing attention in aquaculture. In 2020, global production of cultured algae reached 35.1 million tons (wet weight), accounting for approximately 40% of the total aquaculture production worldwide of 87.5 million tons (FAO, 2022). The dominant species of cultured marine macroalgae are red seaweed (Rhodophyta) and brown seaweed (Heterokontophyta), while green seaweed accounts for less than 1% of global production. However, production is closely monitored for only eight genera of cultured marine macroalgae, which include *Ulva*, *Enteromorpha*, *Caulerpa*, and *Monostroma* (Cai et al., 2021). Among these, *Monostroma* production is only monitored in South Korea with a total output of 6321 tons, accounting for 37% of the total production of green seaweed worldwide. Large‐scale production of *Monostroma* began in Japan more than 60 years ago with annual output ranging from 1400 to 2500 tons of dry product in 1960 (Kida, 1990; Ohno, 1995; Ohno & Triet, 1997). However, *Monostroma* production has not been reported in the World Fisheries Yearbook for many years, possibly because of the close genetic relationships among numerous types of green seaweed, which can easily lead to difficulty in classification (Lewis & McCourt, 2004). For instance, green seaweed of the genera *Ulva* and *Monostroma* are registered in the Food and Agriculture Organization database as “bright green nori” or “green laver,” thus reflecting a certain degree of taxonomic uncertainty (Moreira et al., 2022). In addition, the diversity in external morphology, early development, and reproductive modes has intensified challenges associated with systematic classification and commercial categorization.

Members of the genus *Monostroma* (Chlorophyta, Ulvophyceae) stand out as the most cultivated species of green seaweed with widespread distribution in warm inner bays and estuaries (Bast et al., 2009a, 2009b; Leliaert et al., 2012), and are valued for delicious taste and rich nutritional value (Gupta et al., 2015; Nisizawa et al., 1987; Pise et al., 2012). *Monostroma* polysaccharide extract is widely used in the production of marine medicines, healthcare products, cosmetics, and various industrial applications (Hoang et al., 2015; Karnjanapratum & You, 2011; Lee et al., 2010; Tako, 2017). Japan and South Korea boast the largest cultivation of *Monostroma*, while commercialization endeavors have also commenced in Brazil (Pellizzari & Reis, 2011), India (Kavale et al., 2020), and select regions of China (Chen et al., 2019). Nevertheless, there is no consensus regarding the classification of *Monostroma*. In 1854, Thuret classified all monostromatic green algae (MGA) into the genus *Monostroma* (Ulvaceae) (Thuret, 1854), thus many MGA species were subsequently assigned to this genus. In 1934, Kunieda separated the genus *Monostroma* from the family Ulvaceae based on the life cycle of heterotypic alternation of generations and established the family Monostromaceae (Kunieda, 1934). Since Thuret did not select a type species when establishing *Monostroma*, Monostromaceae was based on the typical life cycle characteristics of *M. latissimum* (Bast, 2015). In view of the polymorphism and life cycle of MGA, scholars have persistently debated the attributes of MGA, leading to prolonged confusion in classification. Based on the presence of intermediate forms of filament, disc, and sac structures during early development of the thallus, as well as considerations of life cycle types, sexual or asexual reproductive modes, classification of MGA has undergone numerous revisions and divisions, resulting in the formation of seven genera (Kaur et al., 2023; Papenfuss, 1960; Tatewaki, 1969): *Monostroma*, *Ulvaria*, *Ulvopsis*, *Kornmannia*, *Protomonostroma*, *Gayralia*, and *Capsosiphon* (Table 1).

**TABLE 1:** Comparison of morphological characteristics among the genera: *Monostroma*, *Ulvopsis*, *Ulvaria*, *Kornmannia*, *Gayralia*, *Protomonostroma*, and *Capsosiphon*.

Genus	Life history	Thallus ontogeny	Zoid release	Zoid flagellum
*Monostroma*	Dimorphic alternation of leafy gametophytes and microscopic sporophytes	Filament‐blade/filament‐sac‐blade	Without pore	Biflagellate gametes, quadriflagellate zoospores
*Ulvopsis*	Dimorphic alternation of leafy gametophytes and microscopic sporophytes, monomorphic asexual	Disc‐sac‐blade	Through pores	Biflagellate gametes and asexual zoids, quadriflagellate zoospores
*Ulvaria*	Isomorphic alternation of generations	Filament‐sac‐blade	Through pores	Biflagellate gametes and quadriflagellate zoospores
*Kornmannia*	Monomorphic asexual sporophytes, Zoospores forming creeping filaments and subsequently releasing zoospores again are also present	Disc‐sac‐blade, Creeping filaments‐ quadriflagellate‐ disc‐sac‐blade	Through pores	Quadriflagellate asexual zoids
*Gayralia*	Monomorphic asexual	Filament‐blade/filament‐sac‐blade	Without pores	Biflagellate zoids
*Protomonostroma*	Dimorphic alternation of leafy sporophytes and microscopic sporophytes	Filament‐blade	Without pores	Quadriflagellate zoids
*Capsosiphon*	Dimorphic alternation of tubular gametophytes and cyst‐like sporophytes			

*Note*: Based on Tatewaki (1969).

Advancements in molecular biology have introduced new techniques for identification and classification of MGA species, including sequencing of the internal transcribed spacer (ITSs) region, *tufA*, *rbcL*, 18S rRNA genes, and randomly amplified polymorphic DNA. In 2009, Bast et al. reported that the early development of *M. latissimum* was characterized by a filament‐blade asexual life cycle (Bast et al., 2009a, 2009b). Analysis of nuclear‐encoded rDNA revealed consistency in the ITS1 sequences of asexual and sexual *M. latissimum*. Therefore, Bast et al. suggested that it was unreasonable to base the genera *Protomonostroma* and *Gayralia* on the typical asexual life cycle, and proposed that members of the genus *Gayralia* should be reclassified to the genus *Monostroma*. In 2013, Pellizzari et al. advocated to classify *M. latissimum* into the genus *Gayralia* along with other species of the genus *Monostroma* (Pellizzari et al., 2013). In fact, macroalgae commonly exhibit both sexual and asexual reproduction (Hiraoka, Dan, et al., 2003). For example, some species of the genus *Ulva* do not undergo normal meiosis or the combination of germ cells, but rather directly complete the life cycle through asexual reproduction (Hiraoka, Shimada, et al., 2003). Species with an asexual life cycle are believed to have lost the ability to undergo sexual differentiation for a second time during evolution (Van Den Hoek et al., 1995), as environmental coercion has been identified as the driving force behind the change in reproductive strategy of *Ulva* species (Ichihara et al., 2019). However, the evolutionary trajectory of sexual and asexual reproduction of MGA species remains unclear.

An earlier study by our group analyzed the external morphological features and ITSs sequences of MGA from various locations along the coast of Guangdong, China (Cui et al., 2022). The results of the phylogenetic analysis revealed that MGA could be categorized into three branches: *Monostroma nitidum*, *Gayralia brasiliensis*, and *Monostroma* sp. The genetic distance between the genera *Monostroma* and *Gayralia* is smaller than the internal genetic distance within the genus *Monostroma* with primary differences in frond thickness and size. However, multigene marker identification and life cycle studies have not been conducted previously. In genome and evolutionary research pertaining to large green algae, the tufA gene and 18S rDNA stand out as prominent molecular marker genes. Studies have shown that the tufA gene, notable for its absence of introns, boasts a high success rate in amplification and demonstrates proficient species identification capabilities, it is deemed suitable as a DNA barcode marker for conducting phylogenetic analyses of large green algae (Saunders & Kucera, 2010). Additionally, 18S rDNA serves as a valuable tool for elucidating the genetic relationships among large green algae and contributes significantly to the establishment of an accurate classification system. It serves as a pivotal data source for inferring the phylogenetic relationships within the realm of green algae (Leliaert et al., 2012). Therefore, the foundations of early research in the laboratory were combined with external morphological and life cycle characteristics, along with *tufA* and 18S rDNA genetic markers of two populations of MGA collected from different habitats to provide theoretical support to elucidate potential evolutionary relationships for the classification of MGA.

## 2 MATERIALS AND METHODS

### 2.1 Collection of MGA samples

**FIGURE 1:**
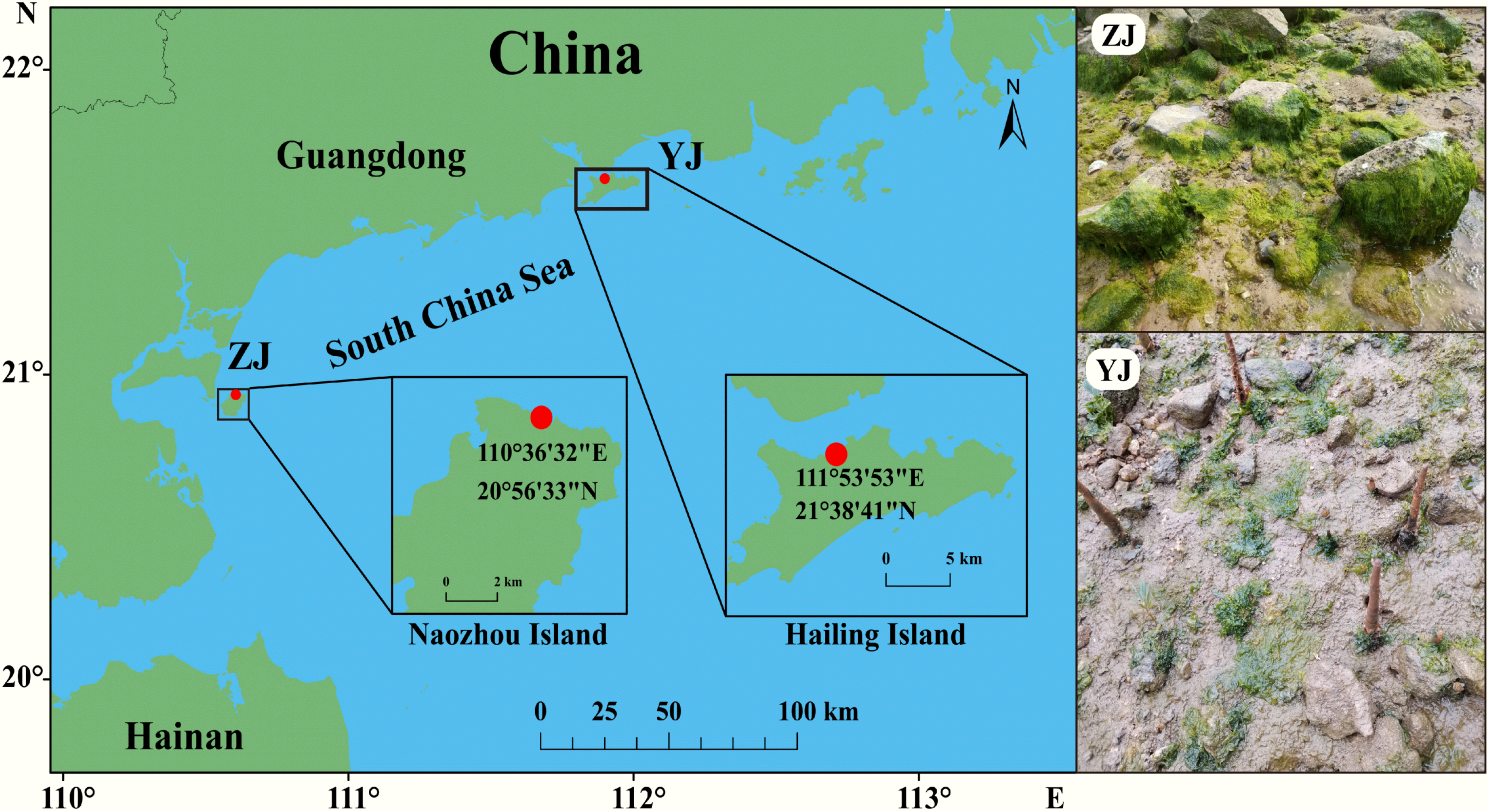
Locations of MGA sample collection and the state of YJ and ZJ samples in the natural habitats.

MGA samples were collected from wild reefs located on Naozhou Island (ZJ; 20°56′33″ N, 110°36′32″ E) and Hailing Island (YJ; 21°38′41″ N, 111°53′53″ E) on March 8 and 11, 2023, respectively (Figure 1). The two sampling sites are both located at the edge of a mangrove. There are no buildings around the ZJ habitat and the substrate is mainly composed of reefs and gravel. The water is highly transparent with a temperature of 24°C, salinity of 32‰, and pH of 8.10. The YJ habitat is located on the edge of a rock dam where a creek enters the sea. The bottom material is mainly sediment and the water is turbid with a temperature of 28.0°C, salinity of 30‰, and pH of 7.84.

Sediments and impurities were removed from the surface of the MGA samples using sterilized seawater and brushes. Subsequently, the samples were temporarily cultured in a light incubator (LRH‐250; Shanghai Yiheng Technology Co., Ltd., Shanghai, China) with a constant temperature of 20°C, light intensity of 20 μmol photons m^−2^ s^−1^, salinity of 30‰, pH of 8.0, and light: dark photoperiod of 12:12 h.

### 2.2 Extraction and sequencing of tufA and 18S rDNA

Total DNA was extracted from three randomly selected YJ samples (YJ‐1, YJ‐2, and YJ‐3) and ZJ samples (ZJ‐1, ZJ‐2, and ZJ‐3) using the Ezup Column Super Plant Genomic DNA Kit (Sangon Biotech (Shanghai) Co., Ltd., Shanghai, China) in accordance with the manufacturer's instructions. Two genes (*tufA* and 18S rRNA) were amplified and sequenced (Lin et al., 2013; Saunders & Kucera, 2010). The universal primer sequences and cited references are detailed in Table 2. Each reaction volume for polymerase chain reaction (PCR) analysis included 1 μL each of the forward and reverse primers (10 μmol/L), 1 μL of DNA template, 1 μL of dNTP Mix (10 mmol/L), 2.5 μL of 10× Taq buffer, and 0.2 μL of Taq DNA polymerase (5 U/μL) with ddH2O added to a total volume of 25 μL. The reaction conditions included a predenaturation step at 95°C for 5 min, followed by 30 cycles of denaturation at 94°C for 30 s, annealing at 63°C for 30 s, and extension at 72°C for 30 s. Subsequently, repair and extension were conducted at 72°C for 10 min, which was followed by a final incubation step at 4°C. The resulting PCR products were separated by electrophoresis with 1% agarose gels in Tris/borate/ethylenediaminetetraacetic acid buffer. The amplified products were sequenced by Shanghai Sangon Biological Engineering Technology & Services Co., Ltd. (Shanghai, China).

**TABLE 2:** Synthetic oligonucleotide primers used for PCR amplification.

Gene	Primer	Sequence(5′ → 3′)	Direction	Reference
*tufA*	tufGF4	GGNGCNGCNCAAATGGAYGG	Forward	Saunders and Kucera (2010)
*tufA*	tufAR	CCTTCNCGAATMGCRAAWCGC	Reverse	Saunders and Kucera (2010)
18S rRNA	NS1	GTAGTCATATGCTTGTCTC	Forward	Lin et al. (2013)
18S rRNA	NS4	CTTCCGTCAATTCCTTTAAG	Reverse	Lin et al. (2013)

Phylogenetic analysis was conducted using Molecular Evolutionary Genetics Analysis (MEGA) software (version 11.0.13; https://www.megasoftware.net/) (Tamura et al., 2021). The *tufA* and 18S rDNA sequences were compared to reference sequences of related taxa retrieved from the GenBank database (https://www.ncbi.nlm.nih.gov/genbank/). Areas of ambiguous alignment were removed. The datasets consisted of 553 and 529 bp. The *tufA* (U09436) and 18S rDNA (JN561078) sequences of Gracilaria lemaneiformis were used as outgroups. Finally, 38 *tufA* sequences and 26 18S rDNA sequences were used for phylogenetic analysis. Evolutionary history was inferred using the neighbor‐joining (NJ) and maximum‐likelihood (ML) methods (Saitou & Nei, 1987) with 1000 bootstrap replicates to assess branch confidence (Felsenstein, 1985). Evolutionary distances were calculated using the maximum composite likelihood model (Tamura et al., 2004) and the units are the number of base substitutions per site. The conserved motifs of the *tufA* and 18S rDNA gene sequences from the samples were predicted using the online motif prediction tool MEME (https://meme‐suite.org/meme/), with the number of motifs searched set to 20.

### 2.3 Morphological observation and cell culture

External morphological observations of 30 randomly selected MGA samples each from YJ and ZJ were conducted. The length, width, and color of the MGA samples were recorded. The MGA samples were manually sectioned with a razor. Micrographs of gametocyte cross sections and surface cells were captured using a microscope (BX53; Olympus Corporation, Tokyo, Japan). Gametocyte thickness and vegetative cell size were measured.

To obtain gametes, the YJ and ZJ samples were cultured separately (temperature, 25°C; light intensity, 60 μmol photons m^−2^ s^−1^; photoperiod, 12‐h light: dark) to induce gametophyte maturation. After about 1 week, visually identifiable gametophyte edge cells transitioned from yellow‐green to yellow‐brown. Microscopic observation revealed the formation of numerous gametocysts. Gametophytes with yellow‐brown edges were collected and dried in the shade at room temperature for 12 h. Then, the gametocytes were stimulated with unilateral light (100 μmol photons m^−2^ s^−1^) to release gametes. Utilizing the phototaxis characteristics of gametes, purified gamete fluid was collected, diluted, and examined for gamete morphology under a microscope. The sex of the gametes was determined by observation of the fusion of gametes from different MGA samples. Finally, the gametes or zygotes were inoculated into a culture dish and subjected to a 12‐h dark: light photoperiod at a constant temperature of 20°C for even deposition and fixation to the dish bottom. Then, the gametes or zygotes were incubated at a constant temperature of 20°C, light intensity of 50 μmol photons m^−2^ s^−1^, and 12‐h light: dark photoperiod. Sterilized seawater was replaced every 3 days. Photographic images were captured regularly to record development.

To obtain zoospores from the ZJ samples, mature sporangia were maintained in the dark for 2 weeks and then stimulated with unilateral light (100 μmol photons m^−2^ s^−1^) to release zoospores. Leveraging the phototaxis characteristics of zoospores, purified zoospore liquid was collected, diluted, and observed under a microscope to identify zoospores morphologically while recording the details. The zoospores were cultivated consistent with the method for culturing gametes or zygotes mentioned above.

### 2.4 Statistical analysis

All data are presented as the mean ± standard deviation. Statistical analyses (one‐way analysis of variance and Duncan's multiple comparison test) were performed using IBM SPSS Statistics for Windows, version 25.0. (IBM Corporation, Armonk, NY, USA). A probability (*p*) value <.05 was considered statistically significant. Graphs were generated using MEGA 11 software (https://www.megasoftware.net/) and Origin 2021 software (https://www.originlab.com/2021).

## 3 RESULTS

### 3.1 Homology analysis based on tufA and 18S rDNA sequences

**FIGURE 2:**
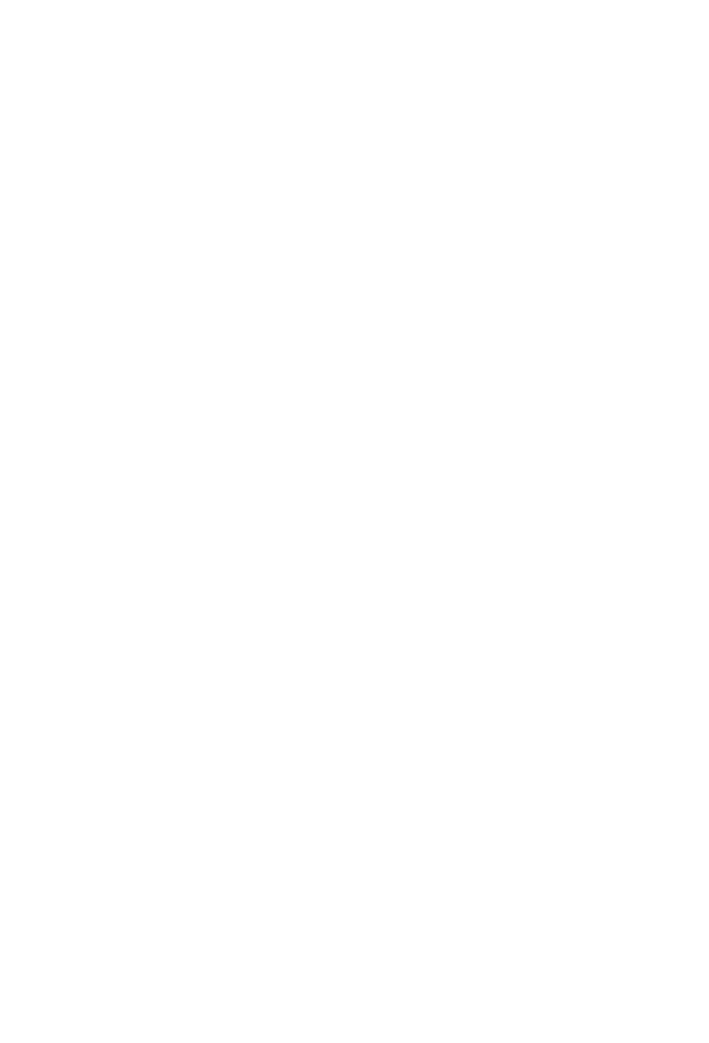
Phylogenetic tree derived from the tufA (a) and 18S rDNA (b) sequences of the YJ and ZJ samples, along with sequences obtained from GenBank, is presented. Experimental samples are highlighted in bold. Bootstrap values from NJ (left) and ML (right) analyses are displayed on the branches. Only bootstrap values exceeding 50% are noted.

All YJ and ZJ samples were identified as *M. nitidum*, as there were no obvious genetic differences. The *tufA* and 18S rDNA sequences of *G. brasiliensis* and *M. nitidum* were homologous. Specifically, the genetic distance between the 18S rRNA gene samples of YJ and ZJ (Y‐18S 1–3, Z‐18S 1–3) is less than 0.0001, and the distance between all such samples and the reference sequence of *M. nitidum* (AF499665) is merely 0.002. For the *tufA* gene samples, the YJ samples (Y‐*tufA* 1–3) exhibit a genetic distance of less than 0.0001 when compared with the reference sequences of *Gayralia* sp. (JF680967) and *G. brasiliensis* (MW242795, OR597303). Similarly, ZJ's *tufA* samples (Z‐*tufA* 1–3) demonstrate a genetic distance of less than 0.0001 with reference sequences of *M. nitidum* (NC072924) and *G. brasiliensis* (NC072923). The genetic distance between the *tufA* samples of YJ and ZJ (Y‐*tufA* 1–3, Z‐*tufA* 1–3) is only 0.005. Additionally, genetic distances between the *M. nitidum* clade and the genera *Kornmannia*, *Ulvopsis*, and *Protomonstroma* range, respectively, between 0.070 ~ 0.074, 0.202 ~ 0.204, and 0.274 ~ 0.279 (Tables 3 and 4). A phylogenetic tree of the samples was constructed based on the *tufA* and 18S rDNA sequences (Figure 2a,b), which included six genera of MGA classified as the Monostromaceae clade and members of the genus *Ulvaria* were classified as the Ulvaceae clade. Within the Monostromaceae clade, the species of *Protomonostroma*, *Capsosiphon*, and *Kornmannia* formed three independent branches that were strongly supported by bootstrap values. Two species classified to the genus *Ulvopsis* (i.e., *M. angicava* and *M. grevillei*) formed an independent branch of the 18S tree and a large branch of the *tufA* tree. All YJ and ZJ samples were clustered in the *M. nitidum* clade for both the *tufA* and the 18S maps. Bootstrap support values were all 100% and the genetic distance between the two was 0.005 and 0.000, respectively.

**TABLE 3:** Genetic distances based on the *tufA* sequences of the YJ and ZJ samples with the reference sequences retrieved from the GenBank.

Species																		
MH308689 *Ulvaria obscura*																		
MZ892916 *U. obscura*	0.005																	
HQ610275 *P. undulatum*	0.278	0.283																
MH475501 *P. undulatum*	0.278	0.283	0.002															
Y‐*tufA*1‐3; JF680967 *Gayralia* sp.; MW242795, OR597303 *G. brasiliensis*	0.282	0.285	0.274	0.276														
MH538598, MH538582, MH538600 *M. grevillei*; MH308479 *Monostroma* sp.	0.251	0.251	0.248	0.247	0.203													
MG944399, MH538555, MF441478 *K. leptoderma*; HQ610252 *G. oxysperma*	0.266	0.269	0.257	0.259	0.074	0.185												
MG646366 *M. angicava*; MK507438, MK507433 *M. hariotii*	0.271	0.271	0.247	0.247	0.202	0.084	0.181											
Z‐*tufA*1‐3; NC072924 *M. nitidum*; NC072923 *G. brasiliensis*	0.288	0.290	0.277	0.279	0.005	0.206	0.070	0.204										
MF544102 *Ulva fasciata*	0.077	0.081	0.297	0.297	0.288	0.265	0.285	0.272	0.293									
MK125445 *U. fasciata*	0.071	0.077	0.297	0.297	0.288	0.268	0.285	0.278	0.293	0.007								
HQ610368 *U. linza*	0.083	0.085	0.285	0.284	0.276	0.253	0.275	0.265	0.281	0.055	0.055							
MW921460 *U. linza*	0.083	0.081	0.298	0.297	0.268	0.248	0.270	0.268	0.273	0.051	0.049	0.036						
MN322753 *U. lactuca*	0.100	0.099	0.320	0.320	0.305	0.267	0.280	0.283	0.308	0.087	0.089	0.113	0.092					
HQ610366 *U. lactuca*	0.102	0.098	0.326	0.325	0.308	0.272	0.286	0.288	0.311	0.091	0.093	0.113	0.092	0.005				
HE600190, HE600188 *U. pertusa*	0.119	0.114	0.318	0.317	0.319	0.288	0.302	0.302	0.325	0.104	0.104	0.119	0.104	0.079	0.075			
MZ870686, KU561325 *U. ohnoi*	0.079	0.083	0.305	0.305	0.288	0.268	0.290	0.275	0.293	0.009	0.009	0.057	0.049	0.095	0.099	0.110		
KC661429 *Enteromorpha ovata*	0.099	0.102	0.290	0.289	0.291	0.269	0.280	0.281	0.297	0.055	0.053	0.061	0.053	0.106	0.106	0.121	0.059	
U09436 *Gracilaria lemaneiformis*	0.382	0.385	0.400	0.400	0.385	0.348	0.378	0.371	0.389	0.397	0.405	0.399	0.396	0.401	0.407	0.421	0.401	0.408

**TABLE 4:** Genetic distances based on the 18S rDNA sequences of the YJ and ZJ samples with the reference sequences retrieved from the GenBank.

Species													
DQ821514 *Capsosiphon groenlandicus*													
DQ821517 *P. undulatum*	0.023												
JF680952, JF680951 *Gayralia* sp.	0.018	0.026											
HQ850570, GU062572 *M. grevillei*, KT180156 *M. angicava*	0.014	0.020	0.020										
MF992130 *M. angicava*	0.014	0.022	0.024	0.004									
Y‐18S1‐3; Z‐18S1‐3	0.018	0.026	0.000	0.020	0.024								
AF499661 *K. leptoderma*	0.050	0.065	0.057	0.055	0.059	0.057							
GU062568, GU062566 *M. kuroshiense*, AF499665 *M. nitidum*	0.016	0.024	0.002	0.018	0.022	0.002	0.054						
AY303590 *Ulvaria obscura*; AB426254 *U. fusca*	0.084	0.099	0.095	0.090	0.090	0.095	0.083	0.092					
DQ286547 *U. fasciata*	0.090	0.097	0.093	0.097	0.101	0.093	0.089	0.091	0.025				
AB425964 *U. fasciata*	0.088	0.095	0.091	0.095	0.099	0.091	0.087	0.088	0.023	0.002			
JN093105, AB425962 *U. linza*	0.084	0.091	0.086	0.090	0.095	0.086	0.083	0.084	0.025	0.006	0.004		
AB425966 *U. intestinalis*	0.084	0.095	0.090	0.090	0.095	0.090	0.087	0.088	0.023	0.010	0.008	0.010	
JN561078 *Gracilaria lemaneiformis*	0.257	0.263	0.263	0.262	0.260	0.263	0.257	0.260	0.267	0.266	0.269	0.266	0.266

Analysis of the *tufA* sequences showed that the YJ samples shared the same sequences as two species of the genus *Gayralia*, *G. brasiliensis* (OR597303, MW242795) and *Gayralia* sp. (JF680967), and the sequences of ZJ samples were the same as those of *M. nitidum* and *G. brasiliensis*. The genetic distance between the YJ and ZJ samples was equal to the intraspecific genetic distance of *Ulvaria obscura* (MH308689, MZ892916) and *Ulva lactuca* (MN322753, HQ610366) (both, 0.005). The model species of *Gayralia*, *G. oxysperma* (HQ610252), shared the same sequence as the *Kornmannia* model species. Additionally, the sequences of *M. angicava* (MG646366) and *M. harriotii* (MK507438, MK507443) were identical. However, *M. harriotii* may have been misidentified here.

**FIGURE 3:**
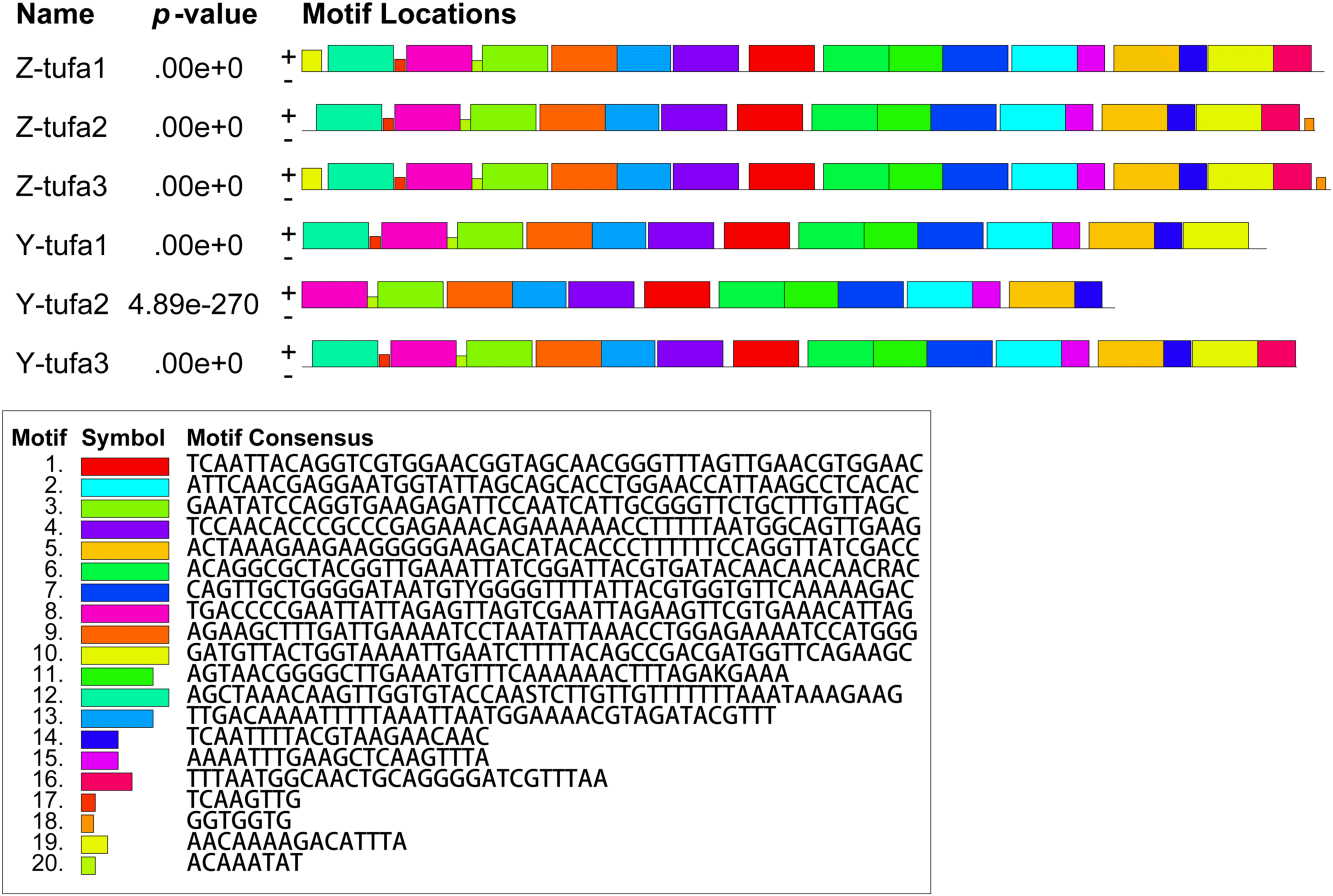
Motif analysis of *tufA* sequences of YJ and ZJ algal strain samples.

**FIGURE 4:**
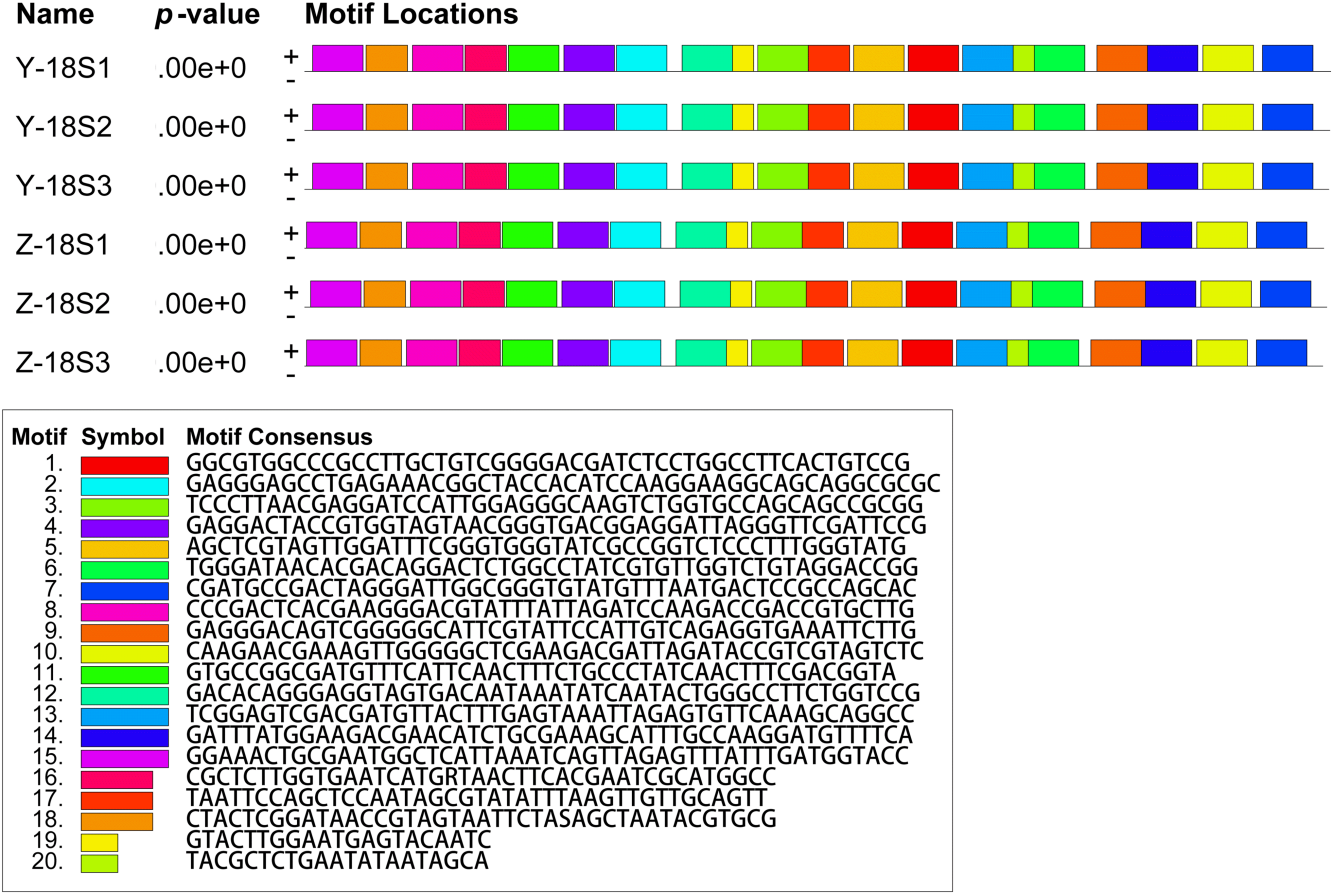
Motif analysis of 18S rDNA sequences of YJ and ZJ algal strain samples.

The 18S rDNA sequences of all ZJ and YJ samples were identical to those of *Gayralia* sp. (JF680952, JF680951) and maintained a genetic distance of 0.002 with *M. nitidum* (AF499665) and *M. kuroshiense* (GU062568, GU062566) within the genus *Monostroma* (*M. kuroshiense* being the collective name for *M. nitidum* and *M. latissimum*, as designated by Bast (2015). The genetic distance between the ZJ and YJ samples was equal to the intraspecific genetic distance of *Ulva fasciata* (DQ286547, AB425964) (0.002), which was smaller than the interspecific genetic distance between *M. grevillei* and *M. angicava* (0.000–0.004) (Tables 3 and 4). Furthermore, the motifs of the *tufA* and 18S rDNA gene sequences were further analyzed through the MME online website, revealing that the *tufA* and 18S rDNA gene sequences are highly conserved, with their distribution and positions on the sequence being essentially consistent (Figures 3 and 4).

### 3.2 Comparative analysis of morphological features

**FIGURE 5:**
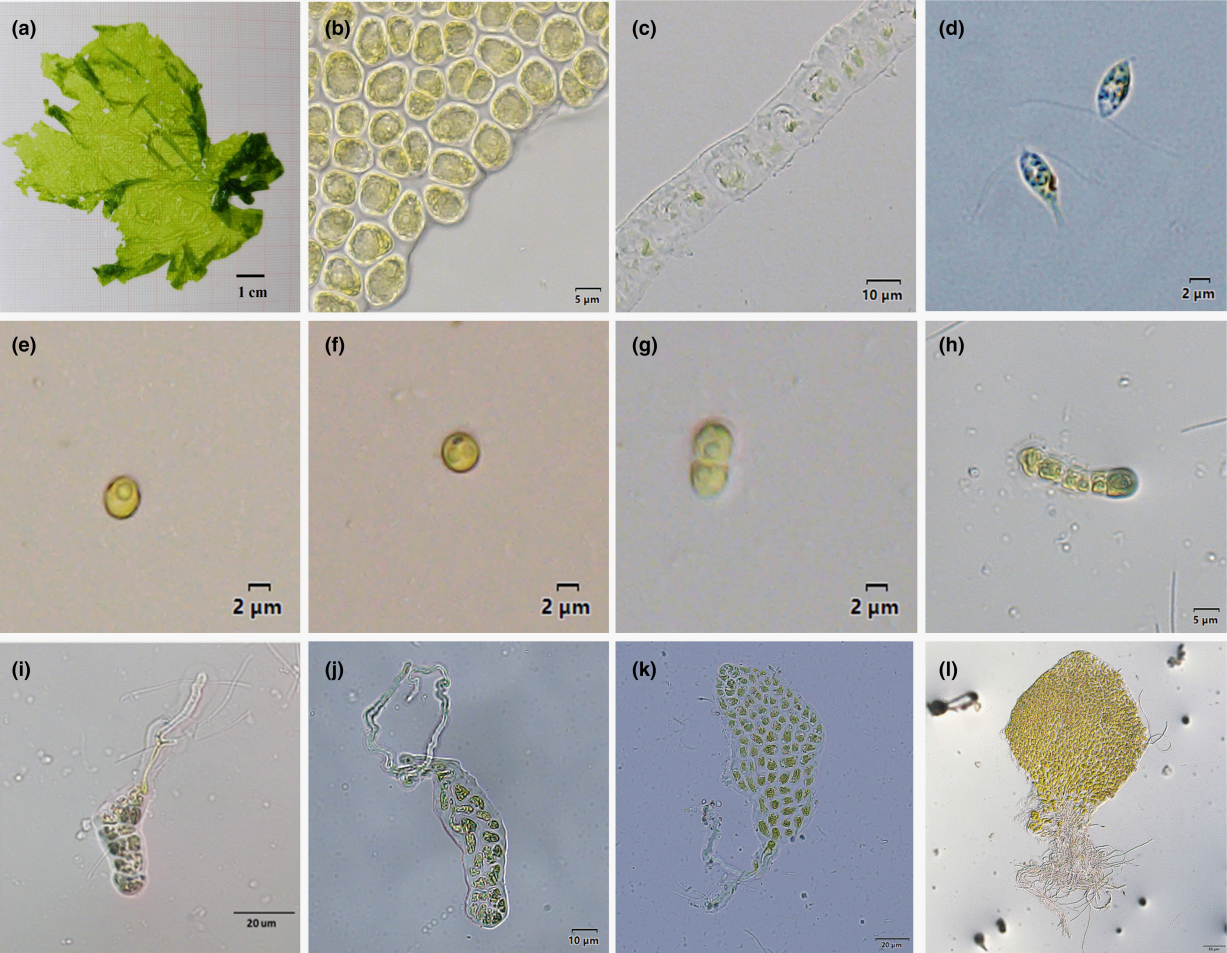
Morphology and ontogeny of the YJ thallus. (a–c), Gametophyte morphology: (a) Gametophyte; (b) Vegetative cells; (c) Gametophyte cross section. (d) Gametes at 1 h after release. (e–l) Germination of gametes: (e) 12 h after release. (f) 24 h after release. (g) 48 h after release. (h) 5 days after release. (i) 17 days after release. (j) 24 days after release. (k) 36 days after release. (l) 60 days after release.

**FIGURE 6:**
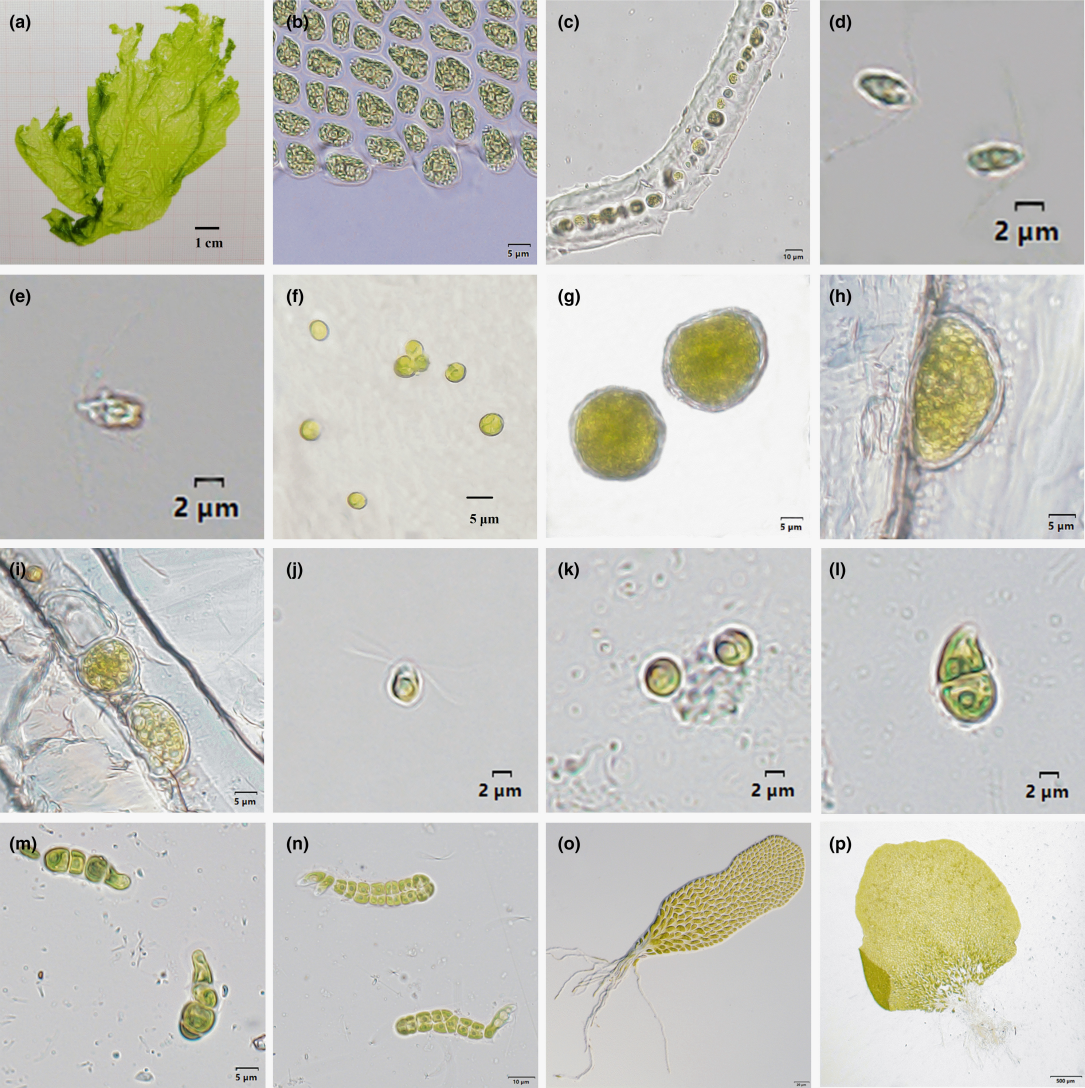
Morphology and ontogeny of the ZJ thallus. (a–c) Gametophyte morphology: (a) Gametophyte; (b) Vegetative cells; (c) Gametophyte cross section. (d) Gametes 1 h after release. (e) Gametes in the process of fusion. (f–j) Formation of sporangia and release of zoospores. (f) Zygotes fixed for 7 days. (g) Sporangia fixed for 180 days. (h) A mature sporangium. (i) Empty sporangia remaining after release of the zoospores. (j) Tetraflagellar zoospores. (k–p) Germination of zoospores: (k) 12 h after release; (l) 24 h after release; (m) 48 h after release; (n) 7 days after release; (o) 24 days after release; (p) 36 days after release.

There were extremely significant differences in morphological features between the YJ and ZJ samples (*p* < .01) (Table 5). The YJ gametophytes were characterized by yellow‐green coloration and smaller dimensions with an average length of 9.21 ± 1.84 cm and width of 7.97 ± 2.23 cm. In contrast, the ZJ gametophytes were light green in color and larger in size with an average length of 36.18 ± 12.22 cm and width of 17.88 ± 5.17 cm (Figures 5a and 6a). Microscopically, the vegetative cells of the YJ and ZJ samples were predominantly arranged in pairs, with distinctly visible nuclei (Figures 5b and 6b). The YJ vegetative cells were round with an average length of 7.11 ± 0.88 μm and width of 5.57 ± 0.81 μm. In contrast, the ZJ vegetative cells were oval with longer dimensions, featuring an average length of 8.37 ± 1.10 μm and width of 4.81 ± 0.85 μm. Notably, the average cross‐section thickness of the YJ samples was approximately half that of the ZJ samples (16.26 ± 1.08 vs. 32.40 ± 2.07 μm, respectively; Figures 5c and 6c).

**TABLE 5:** Morphological characteristics of the YJ and ZY samples.

Sample name	YJ	ZJ
Gamepothyte length (cm)	9.21 ± 1.84^a^	36.18 ± 12.22^b^
Gamepothyte width (cm)	7.97 ± 2.23^a^	17.88 ± 5.17^b^
Vegetative cell length (μm)	7.11 ± 0.88^a^	8.37 ± 1.1^b^
Vegetative cell width (μm)	5.57 ± 0.81^a^	4.81 ± 0.85^b^
Gamepothyte thickness (μm)	16.26 ± 1.08^a^	32.4 ± 2.07^b^
Gamete length (μm)	6.04 ± 0.51^a^	4.1 ± 0.5^b^
Gamete width (μm)	2.42 ± 0.32^a^	2.1 ± 0.32^b^

*Note*: Values are given as mean ± standard deviation (*n* = 30). Means followed by the same letters are not statistically significant according to Duncan test at *p* < .05. Different small letters above the measurements indicate a significant difference between different samples (*p* < .05).

The ZJ gametes were dioecious, while YJ gametes were asexual. Nonetheless, both were collectively released through dissolution and shedding of mature gametocysts at the edges of the gametophytes. There was no trace of the cell wall remaining after the release of the gametes by the gametocysts. Statistically, there were significant differences between the YJ and ZJ gametes (*p* < .01) in both length (6.04 ± 0.51 vs. 4.10 ± 0.50 μm, respectively) and width (2.42 ± 0.32 vs. 2.10 ± 0.32 μm, respectively). The YJ and ZJ gametes had two anterior flagella and exhibited irregular movements after release (Figures 5d and 6d). However, the ZJ gametes moved more rapidly and for longer periods than the YJ gametes. The ZJ gametes exhibited strong positive phototaxis after release but were switched to negative phototaxis after the male and female gametes had combined. Following cessation of movement, the flagella separated and formed a ball. In contrast, the YJ gametes exhibited weak positive phototaxis after release and rapidly shifted to negative phototaxis approximately 5 min later with no observed binding of gametes from different samples.

### 3.3 Life cycle studies

**FIGURE 7:**
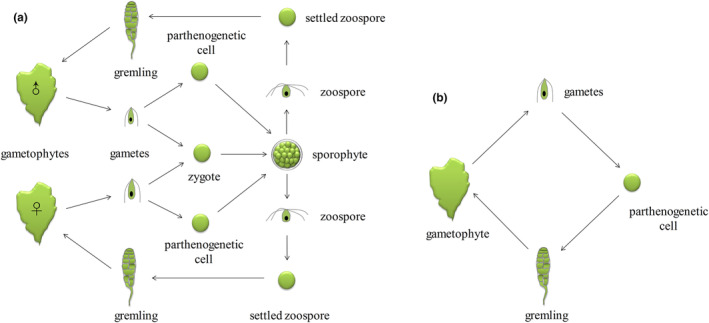
Life history of *M. nitidum*. (a) Sexual or parthenogenesis heterotypic alternation of generation life history. (b) Monomorphic asexual life history.

The life cycle of the YJ samples was monomorphic and asexual (Figure 7b). Gametes appeared as single spherical cells about 3 μm in diameter after 12 h of fixation (Figure 5e). The nuclei began to divide at 24 h (Figure 5f) and formed two cells after 48 h (Figure 5g). However, the process of binary fission was often uneven. The nucleus first moved to one end and divided horizontally upward to form daughter cells, while the mother cell grew downward to form the initial rhizome. At approximately day 5, the gametes developed into uniseriate filament germlings (Figure 5h). On day 17, longitudinal division occurred. Cells in the middle of the uniseriate germlings began to spread toward both ends and a filamentous rhizome became observable at the base (Figure 5i). As longitudinal division progressed, the gametes differentiated into multiple rhizoids by day 24 (Figure 5j) and formed a monolayer of germlings by day 36 (Figure 5k). Ultimately, germlings with diameters of 0.4 mm formed by about day 60 (Figure 5l). Notably, no saccate structures were observed during the early development of gametes. The ontogenetic form of the progeny remained consistent with that of the parents.

The life cycle of the ZJ samples followed the typical sexual dimorphic alternation of generations with the ability to undergo parthenogenesis (Figure 7a). The gametes or zygotes were spherical after fixation (Figure 6f) and underwent slow development and exhibited binary fission. Approximately 2 month later, the gametes or zygotes formed sporangia with cell walls. After fixation for 180 days, the sporangia appeared as round or oval cells with clearly visible cell walls and diameters of approximately 25 μm (Figure 6g). Following 2 weeks of cultivation in the dark, the color of the sporangia transitioned from green to yellow‐green and inner granular zoospores were observed (Figure 6h). In response to intense light stimulation, the zoospores broke through the convex wall of the sporangia and were collectively released. The released sporangia appeared as empty shells (Figure 6i).

The zoospores (mean length, 5.14 ± 0.71 μm; mean width, 3.42 ± 0.57 μm) exhibited strong positive phototaxis and had four superior flagella (Figure 6j). During the early developmental stage, the ZJ zoospores morphologically resembled the YJ gametes with a filament‐blade ontogenetic form but exhibited faster growth. After 12 h of fixation, the zoospores formed spherical cells with diameters of 4 μm (Figure 6k). Unequal division occurred after 24 h, resulting in the formation of two cells (Figure 6l) and uniseriate germlings were observed at 48 h (Figure 6m). Longitudinal division occurred on day 7 (Figure 6n). Around day 24, the zoospores formed a monolayer of germlings (Figure 6o) and eventually matured into germlings with diameters of 2.5 mm by day 36 (Figure 6p). The life cycle of the ZJ offspring and parents was the same.

## 4 DISCUSSION

### 4.1 Recommendations for classification of MGA

**FIGURE 8:**
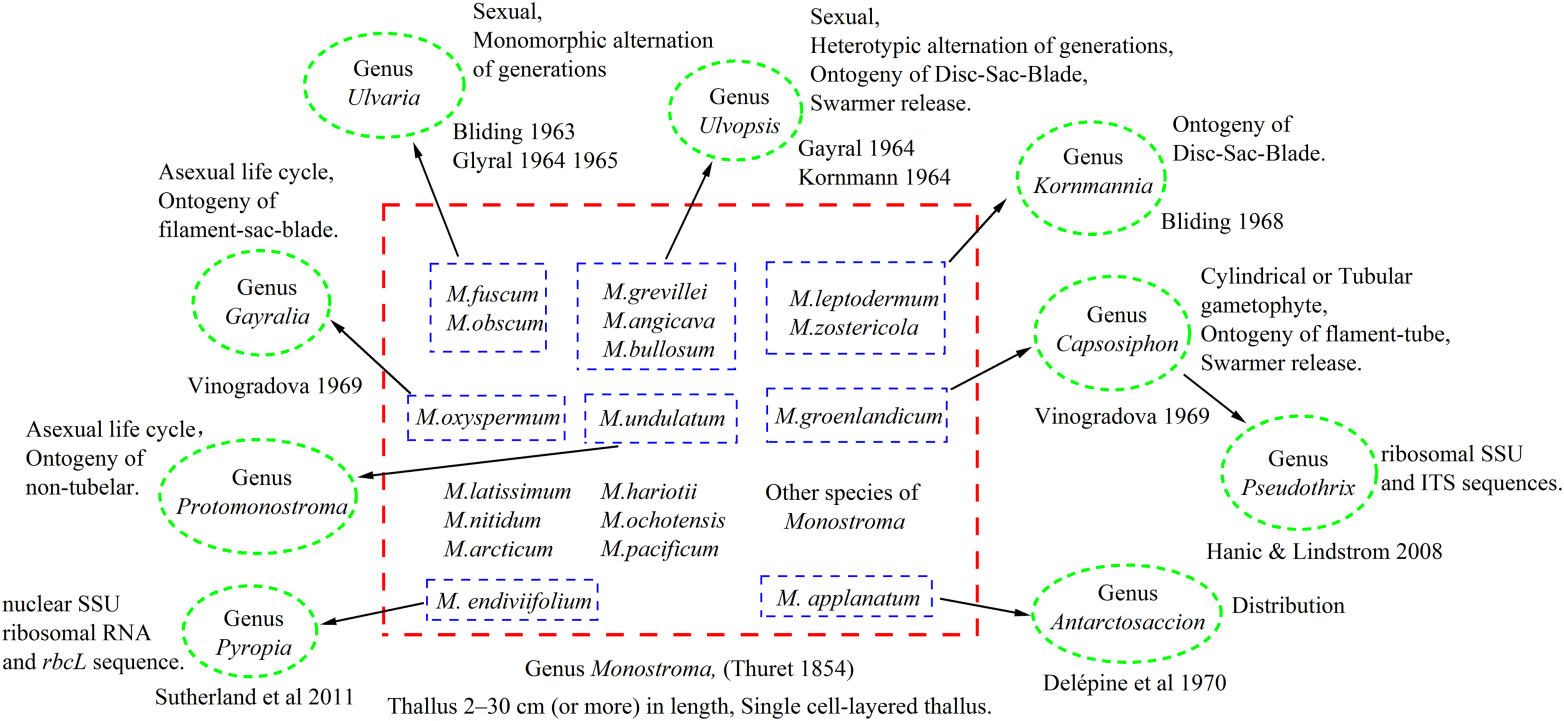
Historical classification of MGA based on morphological and life‐history characteristics. The red dashed box encompasses a subset of species from the genus *Monostroma* during the early stages of family establishment. The green dashed box represents MGA genera considered independently with the indicated classification criteria. The blue dashed box highlights species of the genus *Monostroma* designated to other genera with arrows indicating the reassigned taxa.

The early classification of five genera of MGA (*Ulvaria*, *Ulvopsis*, *Kornmannia*, *Protomonostroma*, and *Capsosiphon*) was reasonably based on morphological and life cycle characteristics. Analyses of the *tufA* and 18S rDNA sequences in this study indicate that the aforementioned five genera formed branches independent of *M. nitidum* (Figure 2a,b), in agreement with the phylogenetic analysis of MGA reported in 2015 by Bast (2015). The genetic distances among these branches based on the *tufA* and 18S rDNA sequences were 0.181–0.283 and 0.014–0.099, respectively (Tables 4 and 5). The genus *Monostroma* established by Thuret in 1854 originally referred to MGA with frond lengths of 2–30 cm, although there was no designated model species. *M. bullosum*, characterized by a heterotypic alternation of generations, and *M. oxyspermum*, displaying monomorphic asexuality, are defined as the original species of the genus *Monostroma* (Bast, 2015; Thuret, 1854). Later studies proposed various classifications of MGA. For example, in the mid‐1960s, Gayral (1964, 1965) advocated for the classification of heteromorphic alternating generations species (*M. grevillei*) within the genus *Monostroma*, which follows a disc‐sac‐blade development pattern in the early life cycle, into the genus *Ulvopsis*. Simultaneously, the monomorphic alternation of generations species (*M. obscurum*) with a filament‐sac‐blade pattern is classified in the genus *Ulvaria*. The genus *Monostroma* would then consist of only the asexual species *M. oxyspermum*, characterized by somatic cells arranged in pairs or fours and the collective release of gametes through the rupture of gametocysts. Kornmann (1964) argued that the early development of disc‐sac‐blade is characteristic of the genus *Monostroma* and the asexually reproduced *M. oxyspermum*, which develops into a filament‐sac‐blade in the early stage, should be excluded from the genus *Monostroma*. From a morphological and anatomical point of view, in 1968, Bliding suggested that *M. zostericola* and *M. leptodermum*, which have microscopic discoid intermediates and leafy sporophytes, should be classified into the new genus *Kornmannia* (Bliding, 1968), although the latter only produces quadrifiagellate asexual zoids. In 1969, Vinogradova classified *M. groenlandicum* into the genus *Capsosiphon* and established two new genera, *Protomonostroma* and *Gayralia* (Vinogradova, 1969), and classified the asexually reproduced species *M. undulatum* and *M. oxysperma* as the model species *Protomonostroma undulatum* and *G. oxysperma*, respectively. However, *C. groenlandicus* has been classified as *Pseudothrix groenlandica* based on the results of a conjoint analysis of ribosomal SSU and ITS sequences (Hanic & Lindstrom, 2008). Additionally, *M. applanatum* and *M. endiviifolium* have been renamed *Antarctosaccion applanatum* (Delépine et al., 1970) and *Pyropia endiviifolia* (Sutherland et al., 2011), respectively (Figure 8).

Hence, asexual life cycle are insufficient as the only classification criteria for genera of MGA, as sexual species also have asexual life cycle.

The genus *Gayralia* was established based on a monomorphic asexual life cycle and the model species *G. oxysperma* had the same *tufA* sequence as three species of the genus *Kornmannia* with a genetic distance of 0.07–0.074 from *G. brasiliensis* in the *M. nitidum* clade (Figure 2a,b). According to Pellizzari et al. (2013) and Cui et al. (2022), the ITS sequences of *G. oxysperma* and *G. brasiliensis* exhibited genetic differences of at least 0.3 and 0.2, respectively. Pellizzari advocated for the retention of the genus *Gayralia* by proposing a new Brazilian species, *G. brasiliensis*. In contrast, Cui, aligning with the perspective of Bast et al. (2009b), argued for the abolition of the genus *Gayralia*. Notably, neither study included species from the genus *Kornmannia* for comparison. In fact, the monomorphic asexual life cycle of *K. leptoderma*, the model species of the genus *Kornmannia*, resembles *G. oxysperma*. The primary distinction is that the former produces quadriflagellate zoids and exhibits discoid structures during ontogeny, while the latter produces biflagellate zoids and lacks discoid structures during ontogeny. A report by Bast (2015) classified the three species *G. oxysperma*, *M. latissimum*, and *M. nitidum* into the same clade based on the ITS1 and ITS2 sequences and advocated for the classification of *G. oxysperma* into the genus *Monostroma*. However, the results of 5.8S sequence analysis did not support this conclusion, as *G. oxysperma* showed no affinity with the two branches of *M. latissimum* and *M. nitidum*. Based on the ITS1 and ITS2 phylogeny trees, *G. oxysperma* and *K. leptoderma* have genetic distances of at least 0.7 and 0.3, respectively, while the 5.8S phylogeny results are similar to those of the present study with *G. oxysperma* showing higher affinity to *K. leptoderma*. Considering the limitations of molecular tools, the present study provides evidence that *M. nitidum* has a monomorphic asexual life cycle, characterized by the production of only asexual biflagellate gametes, which initially exhibited weak positive phototaxis and then quickly changed to negative phototaxis. The early developmental stage was consistent with sexual species and all had a filament‐blade ontogenetic form (Figures 5 and 6). There is no obvious taxonomic difference between the asexual *M. nitidum* and the *G. brasiliensis*, although the latter exhibits positive phototaxis after the release of gametes. The only difference between the two and *G. oxysperma* is the absence of saccate structures in the early developmental stage, the life cycle of *G. oxysperma* is consistent with the parthenogenetic life cycle of *Monostroma wittrockii*. In addition, there are currently only three species in the genus *Gayralia*, including *G. kuroshiense* cultivated in Korea, which was added by Andiska et al. (2023). In fact, *G. kuroshiense* was originally named *M. kuroshiense*, which refers to the collective name of *M. latissimum* and *M. nitidum* (Bast, 2015).

Based on the results of morphological and molecular analyses, we suggest that MGA should be classified into six genera: namely, *Ulvaria*, *Ulvopsis*, *Kornmannia*, *Protomonostroma*, *Capsosiphon*, and *Monostroma*. Following the naming priority principle of the International Code of Nomenclature for algae, fungi, and plants (Turland et al., 2018), *G. oxysperma* and *G. kuroshiense* should retain the original names of *M. oxyspermum* and *M. kuroshiense*, while *G. brasiliensis* should be renamed *M. nitidum*, as all three should be classified to the genus *Monostroma*.

### 4.2 Thoughts on the morphological differences of MGA caused by two habitats

Phenotypic plasticity and genetic variation are important factors in the adaptive evolution of MGA. The emergence of asexual *M. nitidum* is the result of environmental selection, they are secondary and have lost the capacity for sexual reproduction for the second time over a long evolutionary period. In this study, significant disparities were observed in the external morphological characteristics of *M. nitidum* between the YJ and ZJ populations (Table 5), and the life histories exhibit marked distinctions (Figures 5 and 6), as *M. nitidum* of the YJ population only exhibits asexual reproduction. Intriguingly, despite these pronounced differences in morphological and life cycle characteristics, there was no apparent genetic distinction between the two populations, as determined by phylogenetic analysis (Figure 2a,b). The influence of environmental factors on macroalgae morphology (Carrington, 1990; Littler & Littler, 1980) and reproductive strategies (Fierst et al., 2010; Searles, 1980) has been previously reported. Under low‐salinity conditions, MGA exhibit earlier maturation as compared to high‐salinity conditions (Kida, 1990). MGA in well‐sheltered habitats are longer than those in areas with faster currents and larger waves (Bast et al., 2009a). Additionally, sterile conditions can cause abnormal early development (Matsuo et al., 2003). According to a report by Tatewaki (1969) and a study published by O'kelly and Floyd (1984), MGA have 14 life cycle types and none seem to exhibit a single characteristic (e.g., filament, saccate, or discoid intermediate) for differentiation from other genera, which reflects the high degree of phenotypic plasticity of MGA, at least to some extent. The environmental conditions of the YJ habitat seem to be more severe than those of the ZJ habitat. The YJ habitat is located on the edge of the rock dam at an estuary of a creek, thus salinity significantly fluctuates, and the substrate primarily consists of mud and gravel, resulting in relatively high turbidity. This environment is similar to the “geographic parthenogenesis” pattern described by Haag and Verduijn (Haag & Ebert, 2004; Verduijn et al., 2004). In marginal habitats, populations frequently undergo cycles of local extinction and subsequent recolonization. Genetic bottlenecks commonly arise during the recolonization phase, culminating in the production of asexual propagules. Asexual species of many plants and animals are more common in marginal habitats, such as high latitudes or altitudes, where the potential for adaptive evolution is greater (Fagerström & Poore, 2001). Previous reports have documented instances of asexual *M. latissimum* in regions characterized by significant salinity fluctuations (Bast et al., 2009b). *M. latissimum* was previously thought to only exist as a sexual species. Furthermore, the asexual biflagellate zoids produced by *Ulva prolifera* demonstrate a preference for low‐salinity environments (Hiraoka & Higa, 2016). These zoids simultaneously exhibit two mating types genomes and are thought to have evolved through apomeiosis, a process that does not involve chromosome reduction in the sporophyte (Ichihara et al., 2019).

In this study, environmental stress was more severe in the YJ habitat, thus the existence of asexual biflagellate zoids may have resulted from adaptation to more severe environmental fluctuations. Asexual *M. nitidum* may also have evolved in the YJ habitat via apomeiosis. Consequently, asexual *M. nitidum* might be a secondary characteristic that evolved from sexual ancestors. Given a suitable environment, there is potential for asexual *M. nitidum* to re‐evolve into a sexual species. Future research at the genetic level will be instrumental in gaining a deeper understanding of the relationship between reproductive strategies and the evolution of MGA species.

## 5 CONCLUSION

This study employed molecular markers and morphological studies of two populations of *M. nitidum* collected from YJ and ZJ. The results revealed no obvious differences in *tufA* and 18S rDNA sequences between the two populations, although there were extremely significant differences in morphological characteristics. *M. nitidum* collected from YJ exhibited only a monomorphic asexual life cycle, while samples collected from ZJ followed the typical dimorphic alternation of generations. The results of this study demonstrate that an asexual life cycle is insufficient as the only classification of MGA at the genus level. The genus *Gayralia* should be reclassified as *Monostroma*, as MGA includes six genera (*Ulvaria*, *Ulvopsis*, *Kornmannia*, *Protomonostroma*, *Capsosiphon*, and *Monostroma*). In addition, asexual *M. nitidum* resulted from evolutionary preference for incomplete meiosis of the sporophyte. Given a suitable environment, there is potential for asexual *M. nitidum* to re‐evolve sexual reproduction.

## AUTHOR CONTRIBUTIONS

**Jiawei Liao:** Conceptualization (equal); data curation (equal); formal analysis (equal); investigation (equal); methodology (equal); validation (equal); writing – original draft (equal); writing – review and editing (equal). **Sipan Wang:** Formal analysis (equal); methodology (equal); writing – review and editing (equal). **Kun Lin:** Data curation (equal); software (equal). **Yongjian Huang:** Investigation (equal). **Xinyi Chen:** Software (equal). **Rong Xin:** Investigation (equal). **Youyou Guo:** Validation (equal). **Enyi Xie:** Conceptualization (equal); funding acquisition (equal); project administration (equal); supervision (equal); validation (equal); writing – review and editing (equal).

## CONFLICT OF INTEREST STATEMENT

The authors declare no conflict of interest.

## Data Availability

The raw reads of 18S rRNA‐seq and tufA DNA‐seq are deposited in the NCBI GenBank under project accession IDs PP078724–PP078729 and PP084008–PP084013 respectively.
